# Visualization of supramolecular structure of Pluronic F127 micellar hydrogels using cryo-TEM

**DOI:** 10.1016/j.mex.2020.101084

**Published:** 2020-09-28

**Authors:** Laura C.E. da Silva, Antonio C. Borges, Marcelo G. de Oliveira, Marcelo A. de Farias

**Affiliations:** a Institute of Chemistry, University of Campinas, UNICAMP, 13083-970 Campinas, SP, Brazil; b Brazilian Nanotechnology National Laboratory (LNNano), Brazilian Center for Research in Energy and Materials (CNPEM),13083-970 Campinas, SP, Brazil

**Keywords:** Pluronic F127, Concentrated micelles, Automated vitrification system

## Abstract

Pluronic® F127 micellar hydrogels are of growing interest to the biomedical field due to their versatility as drug delivery systems. Pluronic® F127 is a symmetric and amphiphilic triblock copolymer which in aqueous medium self-assembles into micelles that pack togetherwith increasing temperature or concentration, leading to non-flowable hydrogels. The microstructure of these hydrogels is usually investigated by small-angle X-ray scattering, which is not a readily available technique. Conversely, cryo-TEM is a widespread technique used for investigating the morphology of aqueous systems. In the case of Pluronic® F127 micellar systems, the elevated viscosity poses a significant challenge for specimen preparation and, consequently, for cryo-TEM observation. Herein, we show a trustworthy, inexpensive and readily available methodology for preparing specimens of highly viscous micellar solutions and non-flowable hydrogels using an automated vitrification system. With this methodology we were able to visualize not only the morphology of individual Pluronic® F127 micelles -but also the supramolecular structure evolution as a function of concentration. This methodology opens up a wide range of opportunities for hydrogel characterization, although additional systematic studies might be required in order to optimize and replicate it for similar systems.

## Highlights

• Visualization of individual Pluronic® F127 micelles in hydrogels.
• Supramolecular structure evolution of micellar hydrogels as a function of concentration.
• Plunge-freezing of high viscosity solutions.

## Graphical abstract

**Figure:**
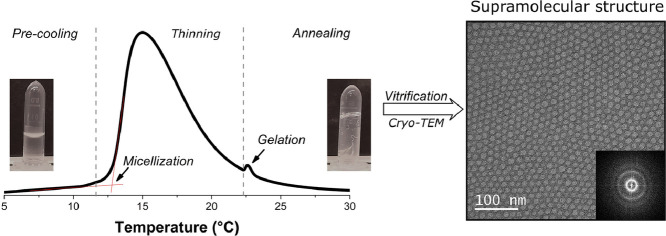

Specifications Table

**Table:** 

Subject Area	Materials Science
More specific subject area	*Electron microscopy; block copolymers*
Method name	*Cryo-TEM specimen preparation of concentrated polymeric micelle solutions*
Name and reference of original method	
Resource availability	Automated Vitrification System

## Method details

### Overview and motivation

Thermoreversible hydrogels of block copolymers (BCP) are widely investigated in the biomedical field. These hydrogels are physically cross-linked by the packing of micelles, which are formed by the self-assembly of the BCP chains [4]. Pluronic® F127, poly(ethylene glycol)-b-poly(propylene glycol)-b-poly(ethylene glycol), is a commercially available amphiphilic BCP that has been extensively investigated as a drug eluting hydrogel due to the possibility of delivering hydrophobic drugs into biological media [2]. This ability is promoted by the presence of hydrophobic poly(propylene glycol) nanodomains (micelle cores) uniformly distributed throughout a poly(ethylene glycol)/water matrix (hydrated micelle corona). While the hydrophobic nanodomains are responsible for incorporating the drugs, the water flow through the matrix is responsible for drug elution and delivery [11].

It is well-known that temperature and concentration are determinant factors for the self-assembly of Pluronic® F127 in aqueous media, as well as its microstructural organization. The critical micelle concentration, cmc, determines the minimum Pluronic® F127 concentration required for the molecules to self-assemble into micelles at a given temperature. In contrast, the minimum temperature required for a Pluronic® F127 aqueous solution to form micelles at a given concentration is named the critical micelle temperature, cmt. Analogously, the critical gelation temperature, cgt, determines the minimum temperature above which the microstructural ordering of micelles results in non-flowable hydrogels.

These reference values (cmc, cmt and cgt) might be significantly altered by the presence of drugs and cosolvents [5]. For this reason, investigating Pluronic® F127 microstructure is key to the development of new drug delivery platforms. Although small-angle X-ray scattering (SAXS) is a model dependent technique, it is currently the most suitable method for determining hydrogel microstructure. Usually, researchers rely solely on the crystallographic knowledge of metals and ceramics to perform peak assignment and microstructure determination [9]. However, when polydisperse polymers or multiple coexisting structures are concerned, multiple conclusions may be possible. In the case of Pluronic® F127 hydrogels, cryogenic Transmission Electron Microscopy (Cryo-TEM) can be used as a valuable resource to corroborate the conclusions drawn from SAXS, but the elevated viscosity of these solutions turns specimen preparation by conventional techniques a challenging task.

To the best of our knowledge, the first TEM micrograph of a Pluronic® F127 hydrogel was reported by Mortensen and Talmon [8] using a 11.7 wt% Pluronic® F127 solution and an in-house developed equipment which they named “Controlled Environment Vitrification System” (CEVS) [3]. A few years later, Lam et al. [6] were the first to report cryo-TEM micrographs of Pluronic® F127 hydrogels prepared using an automatic CEVS, which nowadays is also known as “automated vitrification device” and is widely used for the plunge-freezing of aqueous suspensions. In this latter work, the authors investigated both 5 wt% and 10 wt% Pluronic® F127 solutions. Ordered and well-defined micelles were observed on both concentrations, suggesting that at 50 °C hydrogels of a similar supramolecular arrangement were formed. However, these results do not agree with the well-known Pluronic® F127 phase diagram (Temperature vs wt%) [13], which the authors [6] attributed to a preferential water removal during sample preparation.

Recently, a few authors have reported interesting results using sample preparation methodologies adapted from the work of Lam et al. [6]. First, Pragatheeswaran and Chen [10] imaged 10 wt% Pluronic® F127 hydrogels pure and containing 5 wt% of PEO at distinct molecular weight. The authors used 30 °C, instead of 50 °C, to prepare the specimens and showed that the addition of low molecular weight PEO induced the ordering of micelles. Subsequently, Yom-Tov et al. [14] showed a single cryo-TEM micrograph of a PEO/fibrinogen hydrogel containing 10 wt% of Pluronic® F127 modified to contain acrylate groups. The micrograph was obtained from a specimen prepared at 37 °C and showed well-defined micelles. Both Pragatheeswaran and Chen [10] and Yom-Tov et al. [14] used low Pluronic® F127 concentrations, which formed viscous liquids, while Li et al. [7] imaged a 30 wt% Pluronic® F127 hydrogel, which is a non-flowable hydrogel. Li et al. [7] applied a flowing Pluronic® F127 solution at 4 °C on the TEM grid and allowed it to jellify at 37 °C for 60 s, before vitrification. The cryo-TEM micrograph obtained allowed these authors to calculate the radii of micelles and the interplanar spacings of an fcc arrangement. However, the shape of the micelles was not clearly defined. In our recent work [12], we have used the methodology herein described to image 10 wt% and 20 wt% Pluronic® F127 hydrogels, in which the Pluronic® F127 chain-ends were modified to contain thiol (-SH) groups. Using this methodology, we were able to clearly visualize the morphology of individual micelles within the hydrogels, as well as the distinct supramolecular arrangement of the 10 wt% and 20 wt% Pluronic® F127 hydrogels. In summary, cryo-TEM micrographs of Pluronic® F127 hydrogels are seldom reported and none of the above studies describe the critical experimental details that may allow other authors to obtain reproducible and good quality cryo-TEM images.

Therefore, herein we describe a step-by-step methodology for accurately investigating the morphology of diluted and concentrated Pluronic® F127 solutions as well as non-flowable hydrogels. For the proper microstructural characterization of Pluronic® F127 hydrogels by cryo-TEM, previous rheological and differential scanning calorimetry (DSC) measurements should be performed. Cryo-TEM specimen preparation was conducted using an automated vitrification system (Vitrobot® Mark IV, Thermo Scientific), a benchtop heating oven and a transmission electron microscope (JEOL, JEM 1400Plus), operating at 120 kV, with a OneView 16-Megapixel camera (Gatan).

## Parameters affecting plunge freezing of viscous solutions

Plunge-freezing consists of applying a droplet of the sample on a transmission electron microscope grid, blotting the liquid droplet with filter paper (i.e. removing excess liquid) and plunging it into liquid ethane (i.e. rapid immersion) for vitrification (i.e. amorphous ice formation). Several parameters are variable on an automatic plunge-freezing device, such as blot time, blot force, number of blots and drain time. For most samples, adjusting these parameters is usually enough for obtaining specimens with a suitable ice thickness. However, in the case of the viscous Pluronic® F127 solutions and hydrogels, further adjustments must be made.

Table 1 shows, in chronologic order, the methodologies tested herein. Initially, we tried to reproduce the work of Lam et al. [6]. However, since the 20 wt% samples do not flow through the micropipette tip at 30 °C, we introduced a pre-cooling step and reduced the blot temperature from 50 °C to 37 °C. These alterations resulted in the first method briefly described in Table 1. On this first tentative methodology, extensive investigation of the Vitrobot® parameters were performed, but the results obtained, if any, were not reproducible. As a consequence, we decided to explore other alternatives, which are also briefly described in Table 1. Once we reached our fourth tentative methodology, described in Table 1, the visualization of the micelles and/or the microstructure still seemed random, however, we started to reach fairly reproducible amorphous ice thicknesses. From this point on, efforts were concentrated on optimizing this four-step methodology in order to reach reproducible results. Table 2 shows a summary of the parameters we found to be the most relevant for the plunge-freezing of viscous solutions and hydrogels.

**Table 1:** Specimen preparation procedures tested and their results in terms of amorphous ice thickness and imaging.

Main steps	Amorphous ice thickness	Imaging	Comments
1. Pre-cooling in ice bath. 2. One side blot at 37 – 40 °C and very high humidity. 3. Automatic plunge-freezing	Very thick, with only a few or zero observable regions	Same microstructure irrespective of concentration. No individual micelles were observed	Samples frequently undergo sol-gel transition inside the micropipette tip, impairing specimen preparation
1. Pre-cooling in ice bath 2. Blot at 15 °C 3. Heating to 30 °C during 5 min (drain time) 4. Automatic plunge-freezing	Fairly good, usually about one third of the grid is observable	Usually large unstructured polymer aggregates are seen	The slow cooling of the Vitrobot® chamber leads to long drain time, which causes solid ethane formation on the surface of the liquid ethane. Restricted sol-gel transition.
1. Pre-cooling in ice bath 2. Room temperature 3. Manual blot and plunge	Gel formation on the grid visible to the naked eye	Nothing	Manual blot most often results in premature gelation and no specimens were obtained with enough quality to be inserted on a microscope
1. Pre-cooling at 10 °C. 2. Blot at 15 °C 3. Heating in external oven (45 – 60 °C). 4. Manual plunge	Wide variety of ice thicknesses, all of which are observable	On very thin ice micelles are observed for all concentrations we investigated. Nanostructure observation is widely dependent on ice thickness	Numerous parameters might affect the results. However, most of the time, good quality grids are obtained

**Table 2:** Main parameters affecting specimen preparation, the conditions tested and some comments on the results.

Parameter	Conditions	Comments
**Blot time**	1.5 s to 10 s	For solutions, a total blot time of ≈ 3 s provides the best results. For pastes, a total blot time of 7 to 10 s is preferred
**Number of blots**	1 - 2	If we consider the same total blot time, two blots provide better results than a single blot
**Blot side**	• Sample side covered with plastic foil • Opposite side covered with plastic foil • Both sides with filter paper	Plastic foil wrapping usually promotes irregular ice thickness. Filter paper on both sides is preferred
**Sample volume**	•3 µL •2 µL	For solutions, 2 µL result in some empty areas on the grid. For pastes, 3 µL leads to very thick ice
**Pre-cooling**	• Refrigerator overnight • Ice bath for over 1 h • Ice bath for 10 – 20 min • Controlled temperature (10 – 15 °C)	In general, 15 min at 10 °C provided good results in all cases. The longer the pre-cooling the longer the oven time required for self-assembly
**Blot temperature**	5 °C – 18 °C	For solutions, all conditions were viable For pastes, 15 °C or lower should be used.
Oven temperature	45 °C – 60 °C	See Fig. 1
**Time in oven**	1 min – 5 min	See Fig. 1

**Fig. 1:**
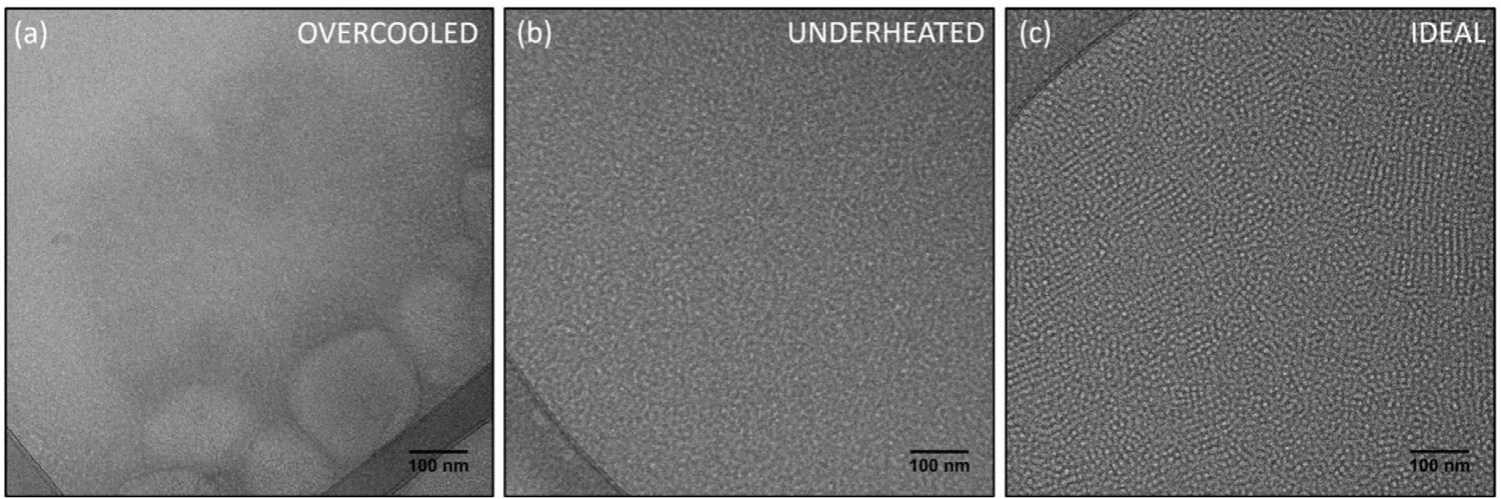
Temperature influence on specimen preparation. TEM micrographs of a 15 wt% sample under (a) prolonged pre-cooling, showing poor self-assembly on the grid; (b) short post-blot heating periods, showing poor micelle packing; (c) ideal conditions (15 min of pre-cooling and 5 min of oven heating), showing well-defined micelles and regions of ordered domains.

In terms of Vitrobot® parameters, the number of blots and the total blot time (i.e. duration) are the most relevant for viscous Pluronic® F127 solutions. The droplet volume applied on the grid is also a relevant parameter because for a fixed volume of the sample the specimen thickness increases with increasing sample viscosity. Nevertheless, since these are thermoreversible hydrogels, the key aspect of this procedure is the temperature control. There are three distinct temperatures to consider; pre-cooling, blot and post-blot, all of which must be carefully selected and controlled. Pre-cooling temperature is the sample equilibrium temperature prior to the application on the grid. Blot temperature is the temperature of the Vitrobot® chamber during sample application and blotting. Finally, post-blot temperature is the temperature of the heating oven in which the sample is left to self-assemble after being removed from the Vitrobot®. Fig. 1 shows representative micrographs that illustrate our conclusions on the temperature control.

At first, we believed that the most critical parameter was the pre-cooling temperature. However, while investigating the pre-cooling temperature range, we verified that prolonged pre-cooling times, even at the highest possible temperature (≈ 18 °C), were as detrimental to the microstructure as shorter periods in excessively low temperatures (≈ 0 °C). In both cases, only amorphous matter was observed, as it is shown in Fig. 1(a). We also found that the post-blot time, rather than temperature, was determinant for reaching the thermodynamic equilibrium of the supramolecular arrangement. Therefore, underheated samples (Fig. 1(b)) could show micelles isotropically distributed, while the samples that were given enough time to reach thermal equilibrium might exhibit some ordered domains (Fig 1(c)). With these findings we concluded that for each experimental step, a time/temperature compromise must be reached in order to allow feasible specimen preparation and reliable morphological observations. While the experimental limitations are good references for selecting the duration of each step, the cmt and cgt values are good references for the temperature selection.

**Fig. 2:**
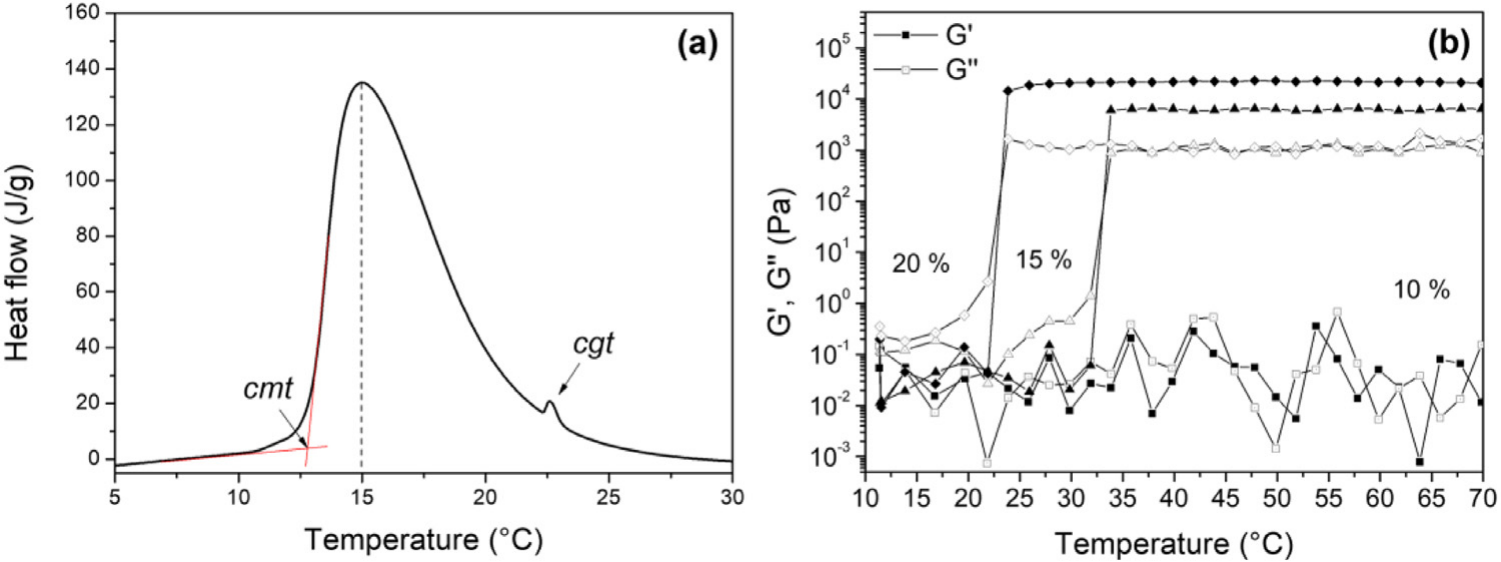
(a) Micro differential scanning calorimetry curve of a 20 wt% Pluronic® F127 hydrogel performed at a 0.5 °C min-1 heating rate in a VP-DSC Microcalorimeter (Microcal Inc). Critical micelle temperature (cmt) and critical gelation temperature (cgt) are indicated by arrows. The dashed line shows the temperature of the highest micellization rate. (b) G' (elastic modulus) and G'' (viscous modulus) of the 10 wt%, 15 wt% and 20 wt% samples obtained from a temperature sweep measured in a Mars III (Thermo Scientific) rheometer. The cgt occurs when G' becomes higher than G'' and is followed by a significant increase of both G' and G''.

The cmt and cgt values of Pluronic® F127 are fairly well-known [1], even so, experimental determination is usually necessary, since the most technologically relevant Pluronic® F127 hydrogels contain drugs, cosolvents or even chain-end modifications. Therefore, as a proof a concept, we used micro differential scanning calorimetry (Fig. 2(a)) and rheological measurements (Fig. 2(b)) to determine the cmt and cgt values of our model system. The micro differential scanning calorimetry curve of Fig. 2(a) shows that the micellization and gelation processes occur sequentially in a wide temperature range of the heating run. The first endothermic peak observed corresponds to the micellization process, with onset at the cmt. However, in dynamic conditions, the maximum rate of micelle formation (dashed line) occur at a temperature higher than cmt, but lower than cgt. Up to this point, the Pluronic® F127 solutions are low viscosity liquids. However, with further heating, the sol-gel transition (gelation) is reached, at the cgt, and a significant viscosity increase is observed. Due to the small enthalpy change that occurs during this process, in some cases the cgt cannot be determined by micro differential scanning calorimetry. Therefore, rheological measurements may be required. On a temperature sweep experiment, such as the one shown in Fig. 2(b), the cmt is not observed, since the solution remains a low viscosity liquid upon micelle formation. In contrast, the sol-gel transition, or cgt, is easily found at the point where G’ becomes higher than G’’, which causes a sharp increase of both parameters, due to the viscosity increase.

If we consider that the thermal equilibrium is always reached within the timeframe of the experiment, ideally, the pre-cooling temperature and the blot temperature should be below the cmt, to allow the sample to flow through the micropipette, as well as to prevent the preferential removal of water from the absorption with filter paper. The post-blot temperature, on the other hand, should be above the cgt, in order to allow the in situ micellization and gelation. Nevertheless, if the sample is initially equilibrated at a temperature much lower than the cmt, it may not form micelles or gel within the timeframe of the specimen preparation, due to insufficient time for thermal equilibrium. In this sense, the best approach is to reduce the temperature interval of the experiment, so that a few minutes are enough for temperature equilibration.

As an example, for the 20 wt% sample shown in Fig. 2(b), 10 °C is an ideal pre-cooling temperature, since it is just below the cmt, where the sample flows easily through the micropipette tip and is easily absorbed by the filter paper. Since this is the first step, there are no limitations concerning its duration. In contrast, the following step (blot, Table 1) does not take more than 30 s to conclude. Therefore, it is fair to assume that blot temperature should not have a profound influence on the self-assembly behavior. However, it was verified that blot temperatures above the cgt might result in early gelation, inside the micropipette tip, if Pluronic® F127 concentration is sufficiently high. Therefore, the temperature of maximum micelle formation, 15 °C, should be a suitable alternative, inputting a mild heating rate to the system, which might allow micellization to begin simultaneously with the blot.

Even though the cgt value is a quantifiable parameter for controlling the supramolecular arrangement of the hydrogel, the selection of the post-blot temperature should also take into account the desired application of the material under investigation, since the hydrogel microstructure is severely dependent on the working temperature. As it is illustrated in Fig. 2(b), if the material is being developed to be handled at room temperature (≈ 25 °C), 10 wt% and 15 wt% Pluronic® F127 solutions would be in the sol state, presenting themselves as low viscosity liquids, while the 20 wt% Pluronic® F127 solution would be in the gel state, which is in fact a paste rather than a solution. Therefore, at 25 °C isotropically distributed micelles would be found on the 10 wt% and 15 wt% samples, while ordered micelles would be found on the 20 wt% sample. Conversely, if the material is to be used in biomedical applications, physiological conditions (≈37 °C) would be more suitable. In this case, the 15 wt% sample should present a morphology more closely related to the 20 wt% than the 10 wt%, due to fact that G’ is now higher than G’’ and significantly higher than G’ and G’’ at 25 °C, as shown in Fig. 2 (right). Finally, it is also noteworthy mentioning that in some cases it might be more interesting to select distinct temperatures for each sample, to measure micelle size on the gel state as a function of concentration for example.

**Fig. 3:**
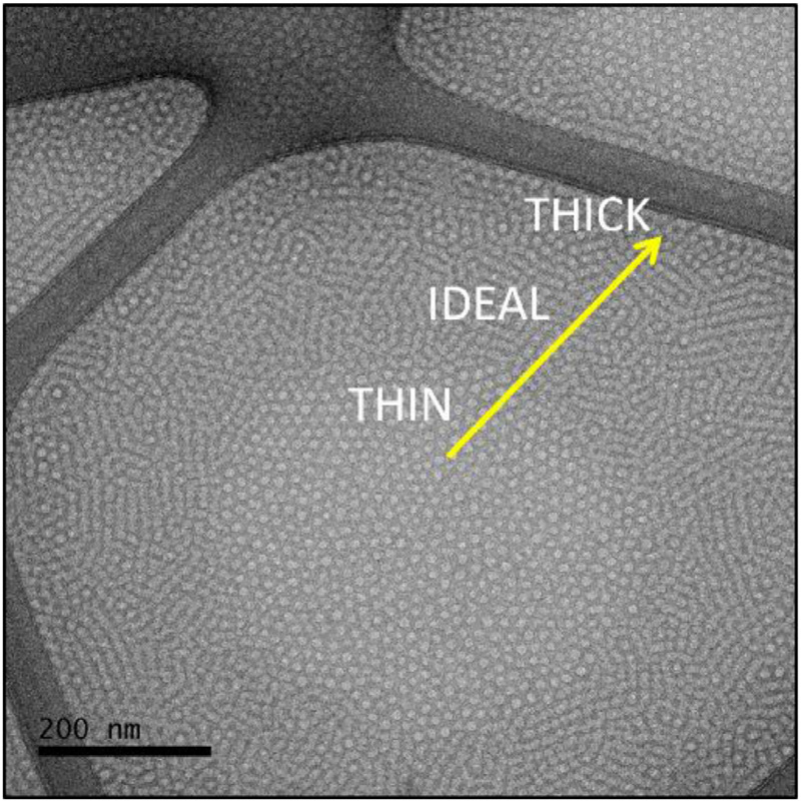
Ice thickness influence on nanostructure observation.

Once the temperature and duration of each experimental step has been carefully considered and suitable specimens are obtained, it is important to consider the ideal amorphous ice thickness for imaging. Although there is a wide ice thickness range in which a specimen could be suitable for analysis, not all thicknesses provide the same results, as it is shown in Fig. 3. If the sample is too thin, a monolayer of micelles is observed, which is a very good condition for micelle size studies. In contrast, for the nanostructure investigation, a medium ice thickness could be preferred, to allow a few layers of micelles to be imaged together, evidencing micelle packing. However, there is an ice thickness limit above which the overlapping of several layers of micelles impairs visualization.

**Fig. 4:**
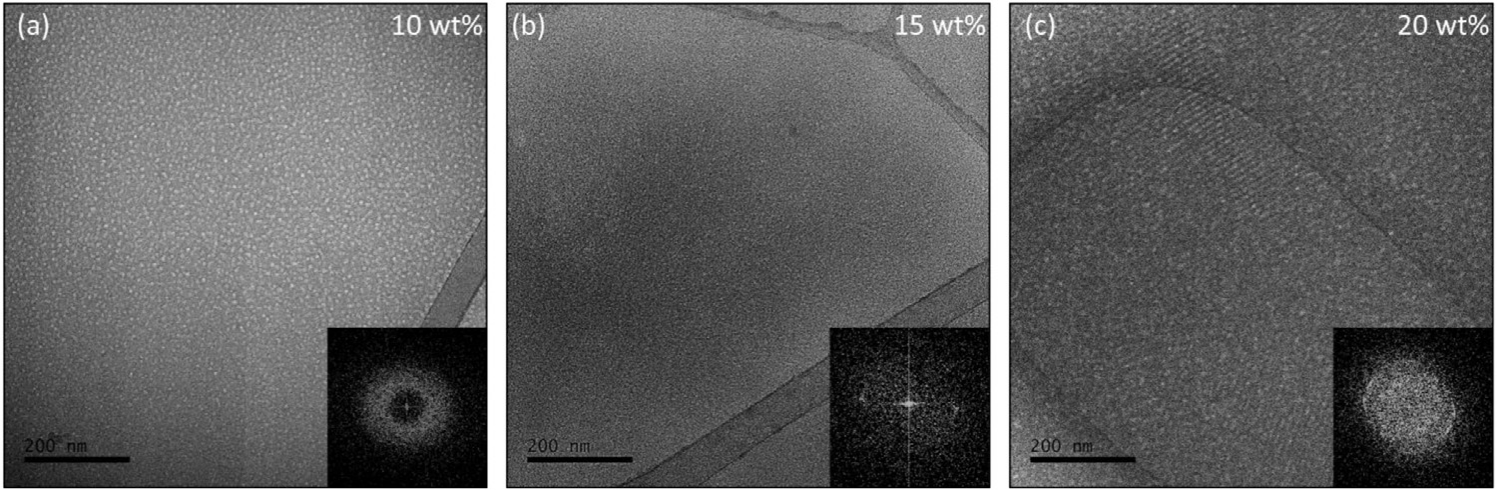
Method validation. (a) 10 wt%, (b) 15 wt% and (c) 20 wt% samples were prepared under the exact same conditions in order to observe the morphological evolution as a function of concentration of Pluronic® F127 solutions at 55 °C.

The superposition of ordered layers of micelles results in micelle rows that are clearly distinguished on the regions of intermediary ice thickness (Fig. 3) and bring information on the extent of the ordered domains. To the naked eye, these micelle rows might not bring much information, since the supramolecular arrangement is not evident and individual micelles are not clearly distinguished. In this case, the fast Fourier transform (FFT) is a useful tool, as it is shown in Fig. 4, which is a validation of this method. We used a pre-cooling temperature of 10 °C, a blot temperature of 15 °C and a post-blot temperature of 55 °C to prepare specimens of 10 wt%, 15 wt% and 20 wt% Pluronic® F127 solution. Based on the results shown in Fig. 2(b) it would be expected that micelle rows would be observed in the 15 wt% and 20 wt% samples, while the 10 wt% sample would exhibit isotropically oriented micelles. The micrographs shown in Fig. 4, as well as their respective FFT, corroborate these assumptions and validate our protocol.

The isotropic distribution of micelles at the 10 wt% sample (Fig. 4(a) is clearly verified and in agreement with the rheological measurements of Fig 2(b) as well as the results earlier described by Pragatheeswaran and Chen [10]. Conversely, micrometer sized domains of micelle rows are clearly observed at the 20 wt% sample (Fig. 4(c)) and, even though these domains are also present at the 15 wt% sample (Fig. 4(b)), in this case contrast is very low. The supramolecular structure observed for the 20 wt% sample (Fig. 4(c)) is equal to the supramolecular structure shown by Li et al [7] for the 30 wt% Pluronic® F127 hydrogel, which is due to the fact that the authors used a methodology apparently similar to ours, and is in agreement with the well-known Pluronic® F127 phase diagram [13]. Finally, the morphological evolution as a function of Pluronic® F127 concentration is clearly observed herein from the FFT (insets of Fig. 4), which is isotropic for 10 wt%, mostly isotropic with well-defined spots related to the existence of oriented regions at the 15 wt% and, finally, multiple oriented regions isotropically oriented that result in a ring on the FFT of the 20 wt% sample. It is noteworthy mentioning that we chose to keep all parameters constant and to prepare the samples sequentially to ensure the same thermal history to all samples, since our intent was to validate the method through the accurate determination of the morphological evolution. However, at this temperature the viscosity of the 15 wt% and 20 wt% is extremely high, which impaired both the thermal diffusion and the achievement of ideal ice thicknessess. Therefore, one might achieve better results, such the ones shown in Fig. 3, by increasing both the pre-cooling and the post-blot times.

## Optimized procedure (Timing 40 min)

**Step 1.** Initially, the Vitrobot® chamber was set at 10 °C (See Fig 2) and 100% humidity and left to equilibrate.**Caution:** Vitrobot® water reservoir should be filled beforehand.

**Step 2.** Vitrobot® parameters were adjusted: “blot time” was set at 3.5 s, “blot force” at 0 and “number of blots” at 2. Both the “blot wait” and the “drain time” parameters were set at zero. **Note:**blot force should be calibrated for each equipment. Blot time could be variable from one sample to another. The less viscous the sample is, the less time it is required.

**Step 3.** The benchtop heating oven was set at 55 °C and a closed Petri dish containing moisturized gaze was placed inside the heating oven for equilibrating both temperature and humidity.

**Fig. 5:**
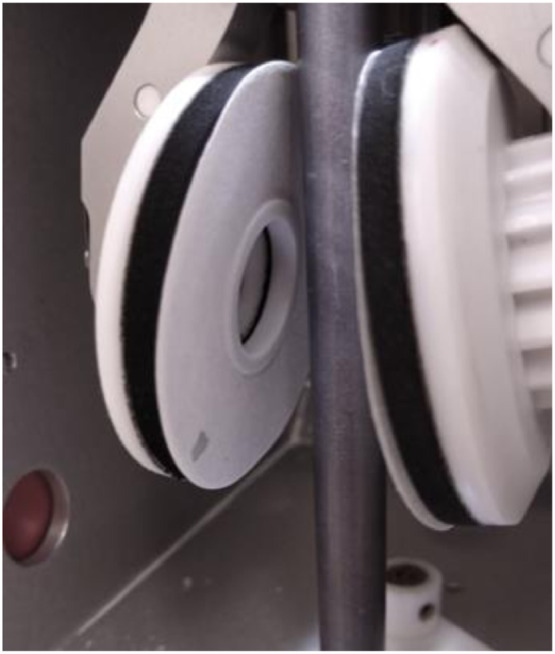
Photograph of the blot mark on the filter paper placed on the opposite side from sample application.

**Step 4.** Lacey TEM grids (Prod. # 01885-F, Ted Pella) were placed on a microscope slide covered with Parafilm®, which was subsequently placed inside the glow discharge system (easiGlow, Pelco) and glow discharged at 0.37 mbar and 25 mA for 50 s. Note: Glow discharging should be performed a few minutes before sample preparation. A good parameter to observe if glow discharging is efficient is the blot side. After blotting, the filter paper on the opposite side of sample application should be wet. See Fig. 5.

**Step 5.** Liquid ethane was prepared by blowing gaseous ethane onto a container filled with liquid nitrogen. **Caution:**This step should be carried out on a fume hood since ethane is an asphyxiating gas. Liquid nitrogen temperature is also a cold burning hazard.

**Step 6.** Samples were pre-cooled at 10 °C for 15 min. **Note:**Pre-cooling is required to allow the sample to flow through the micropipette tip. However, prolonged cooling periods might impair analysis. See Fig 1.

**Fig. 6:**
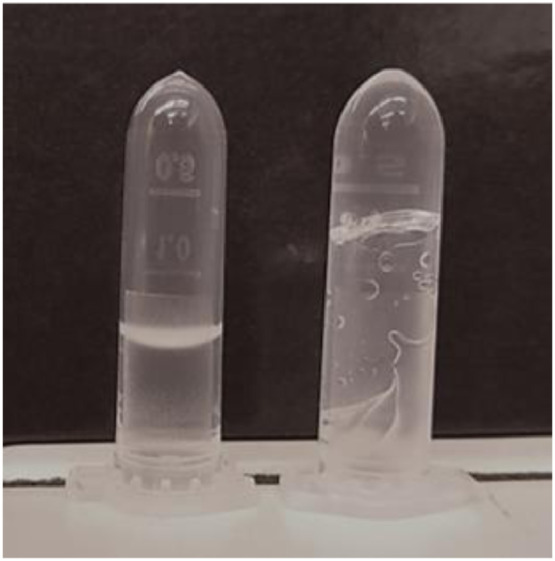
Viscosity assessment. On the left, a diluted sample of low viscosity (3 µL drop is preferred). On the right, a concentrated sample of high viscosity (2 µL is preferred).

**Step 7.** A glow discharged TEM grid was placed inside the Vitrobot® chamber and a 2 – 3 µL drop of the sample was placed directly onto the carbon side of the grid with the aid of a micropipette. **Note:**2 µL drops are preferred for samples that at room temperature are non-flowing hydrogels, or pastes, while 3 µL are preferred for the samples that flow at room temperature. See Fig. 6.

**Fig. 7:**
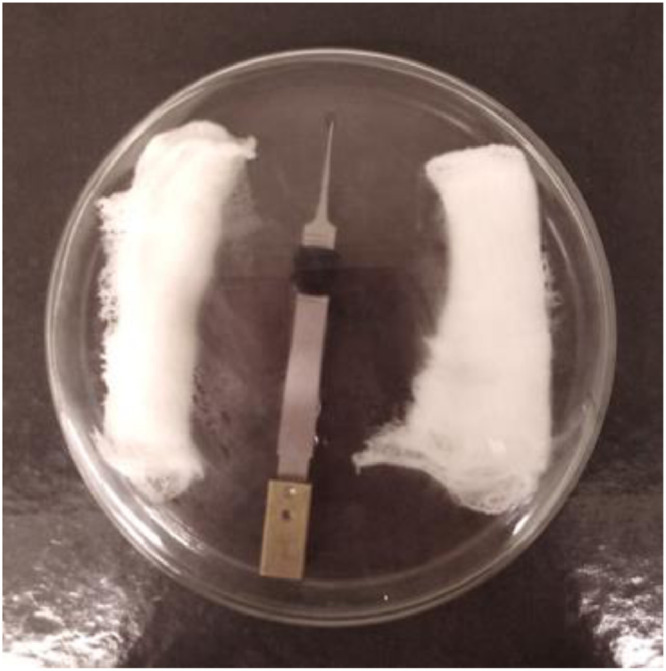
Photograph of the Petri dish used for the post-blot heating at 100% humidity.

**Step 8.** Blotting was performed immediately after sample application, in the absence of a liquid ethane container on the bottom of the Vitrobot® chamber. After blotting, the equipment plunges the sample automatically, by rapidly lowering the tweezer containing the grid. However, since the liquid ethane container was not placed on the bottom of the chamber, plunging merely exposed the tweezer to the atmosphere. The tweezer was then readily removed from the Vitrobot^Ⓡ^ and placed immediately inside the heated Petri dish (Fig. 7), which in turn was placed back inside the benchtop heating oven. Note: Once the grid exits the Vitrobot® chamber it starts to dry rapidly due to the non-saturated atmosphere (i.e. air humidity <100%). Therefore, this is a time sensitive step and it is advisable that two people are involved.

**Step 9.** Sample was left to self-assemble for 5 min at 55 °C and 100% humidity. Subsequently the specimen was manually plunged into liquid ethane and stored in liquid nitrogen. **Note:**We found that 5 min was the best compromise between equilibrating temperature and preventing liquid ethane contamination when three samples were prepared sequentially. The decision of using the benchtop oven instead of the drain time parameter of the Vitrobot® was made based on the fact that the slow heating rate of the equipment led to the formation of a thin layer of solid ethane on the surface of the liquid ethane during the drain time.

**Fig. 8:**
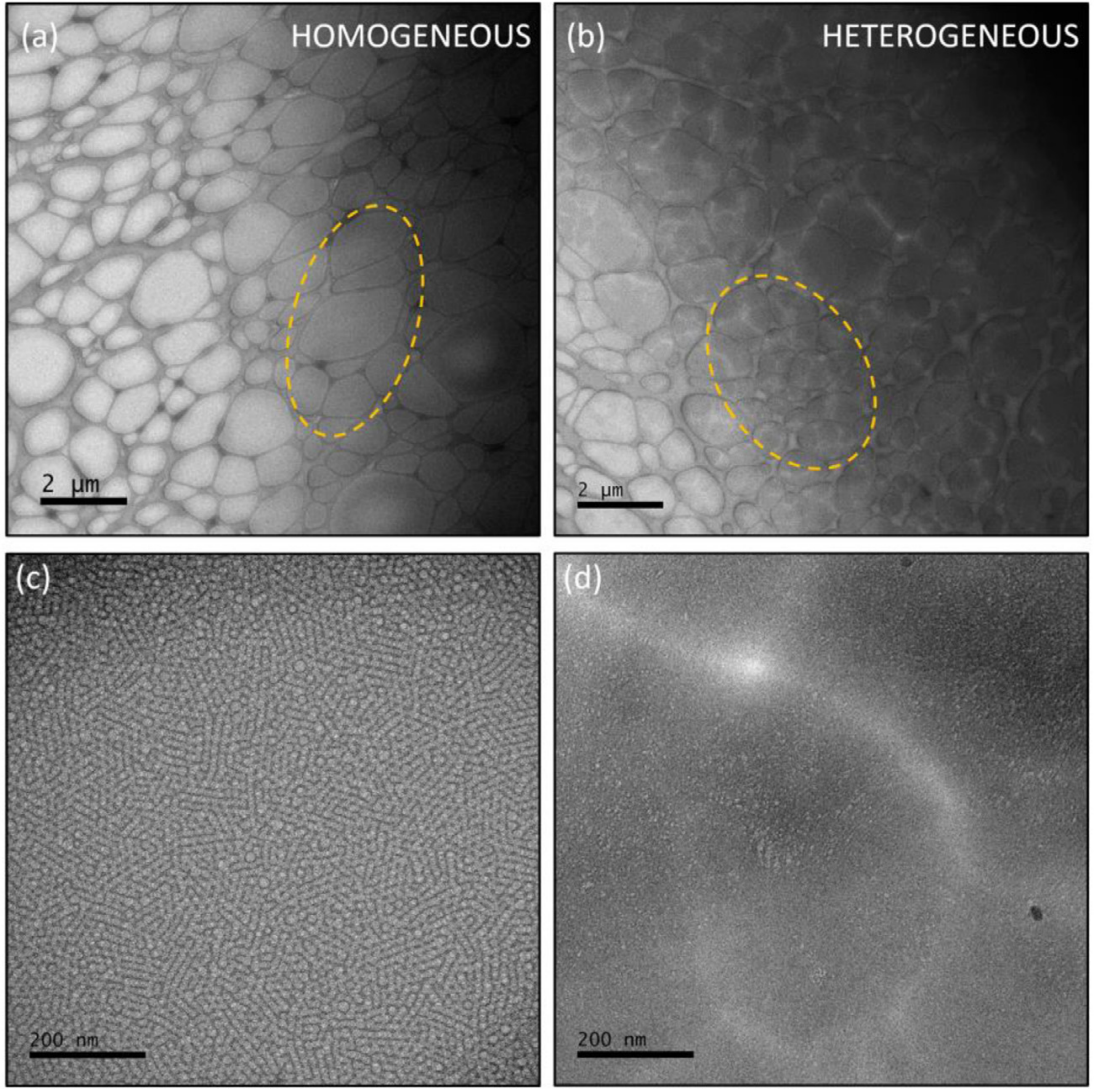
Ice thickness guidelines. (a) and (b) show low magnification micrographs. The ideal ice thickness is highlighted by the yellow dashed lines in (a), while the yellow dashed lines marked in (b) highlight a region of approximately the same ice thickness as the one marked in (a), however, in a heterogeneous region of the grid. Possibly, the heterogeneous regions are due to the aggregation of amorphous matter that has not yet underwent self-assembly. (c) and (d) show representative higher magnification micrographs of the highlighted regions of (a) and (b), respectively.

Step 10. Sample observation must be conducted on a microscope in the 200 – 80 kV range, operating in low dose conditions. If sample height is carefully adjusted, a ≈ -1.5 µm defocus should be enough. Homogeneous ice of medium thickness should provide the best results. See Fig. 8 for an example.

## Conclusion

We have developed a new methodology for preparing cryo-TEM specimens of Pluronic® F127 micellar hydrogels using an automated plunge-freezing device (Vitrobot®, Thermo Scientific). Two important issues were addressed: how to obtain specimens that are sufficiently thin for imaging, in spite of the elevated viscosity of the samples and how to ensure that the supramolecular structure observed was not an artifact of sample preparation. We concluded that the critical gel temperature is a key parameter to be considered. In terms of specimen ice thickness, the best results are obtained when the sample is pre-cooled to a temperature just below the cmt, where the sample is a low viscosity liquid, and blotted at a temperature above the cmt and below the cgt. Afterwards, enough time should be given for the micellization and/or gelation to occur directly on the grid at a pre-determined temperature, prior to manually plunging it into liquid ethane. More viscous samples require more time for self-assembly. However, if multiple samples must be prepared sequentially, post-blot heating should not surpass 5 min, in order to reduce liquid ethane contamination. In terms of microstructure observation, homogeneous regions of intermediary thickness provide the best results. We believe that this protocol is a suitable alternative for imaging micellar hydrogels and it might be easily replicated on any cryo-TEM facility.

## Declaration of Competing Interest

The authors declare that they have no known competing financial interests or personal relationships that could have appeared to influence the work reported in this paper.
